# Metagenomic Characterization of Gut Microbiota in Individuals with Low Cardiovascular Risk

**DOI:** 10.3390/jcm14145097

**Published:** 2025-07-17

**Authors:** Argul Issilbayeva, Samat Kozhakhmetov, Zharkyn Jarmukhanov, Elizaveta Vinogradova, Nurislam Mukhanbetzhanov, Assel Meiramova, Yelena Rib, Tatyana Ivanova-Razumova, Gulzhan Myrzakhmetova, Saltanat Andossova, Ayazhan Zeinoldina, Malika Kuantkhan, Bayan Ainabekova, Makhabbat Bekbossynova, Almagul Kushugulova

**Affiliations:** 1 National Laboratory Astana, Center for Life Sciences, Nazarbayev University, Astana 010000, Kazakhstan; 2 Department of Internal Medicine with the Courses of Gastroenterology, Endocrinology and Pulmonology, NpJSC Astana Medical University, Astana 010000, Kazakhstan; 3 Heart Center, Corporate Fund “University Medical Center”, Nazarbayev University, Astana 010000, Kazakhstan

**Keywords:** low cardiovascular risk, gut microbiome, shotgun sequencing

## Abstract

**Background/Objectives:** Cardiovascular diseases remain the leading cause of global mortality, with the gut microbiome emerging as a critical factor. This study aimed to characterize gut microbiome composition and metabolic pathways in individuals with low cardiovascular risk (LCR) compared to healthy controls to reveal insights into early disease shifts. **Methods:** We performed shotgun metagenomic sequencing on fecal samples from 25 LCR individuals and 25 matched healthy controls. Participants underwent a comprehensive cardiovascular evaluation. Taxonomic classification used MetaPhlAn 4, and functional profiling employed HUMAnN 3. **Results:** Despite similar alpha diversity, significant differences in bacterial community structure were observed between groups (PERMANOVA, *p* < 0.05). The LCR group showed enrichment of *Faecalibacterium prausnitzii* (*p* = 0.035), negatively correlating with atherogenic markers, including ApoB (r = −0.3, *p* = 0.025). Conversely, *Fusicatenibacter saccharivorans* positively correlated with ApoB (r = 0.4, *p* = 0.006). Metabolic pathway analysis revealed upregulation of nucleotide biosynthesis, glycolysis, and sugar degradation pathways in the LCR group, suggesting altered metabolic activity. **Conclusions:** We identified distinct gut microbiome signatures in LCR individuals that may represent early alterations associated with cardiovascular disease development. The opposing correlations between *F. prausnitzii* and *F. saccharivorans* with lipid parameters highlight their potential roles in cardiometabolic health. These findings suggest gut microbiome signatures may serve as indicators of early metabolic dysregulation preceding clinically significant cardiovascular disease.

## 1. Introduction

Cardiovascular diseases (CVDs) remain the leading cause of mortality and morbidity worldwide, accounting for approximately one-third of all deaths globally [1,2]. While traditional risk factors such as hypertension, dyslipidemia, diabetes, and smoking have been well established, they are not enough to thoroughly explain the incidence and progression of CVDs [3,4]. Recent advances in research have highlighted the potential role of the gut microbiome in cardiovascular health [4], suggesting a complex interplay between microbial communities and host metabolism [5].

The human gut microbiome, comprising trillions of microorganisms, has emerged as a critical modulator of various physiological processes. Growing evidence indicates that alterations in gut microbial composition and function may contribute to several pathological conditions, including atherosclerosis, hypertension, heart failure, and thrombosis [6,7]. These alterations can influence cardiovascular health through multiple mechanisms, such as the production of metabolites that affect host physiology, modulation of systemic inflammation, and regulation of metabolic pathways linked to cardiovascular risk factors [8]. Moreover, microbiota can influence cardiovascular risk, both in a potentially atherogenic and protective capacity, suggesting opportunities for personalized treatment [9].

Most studies investigating the relationship between gut microbiome and cardiovascular health have focused on patients with established CVD or those with high cardiovascular risk profiles [9,10,11,12,13]. However, the temporal relationship between microbiome alterations and CVD development remains poorly understood. Specifically, it is unclear whether changes in the gut microbiome occur early in the pathogenesis of CVD, potentially preceding the clinical manifestation of the disease.

Thus, the concept of a “cardiovascular disease continuum” recognizes that cardiovascular disorders develop progressively over time, with an individual’s position along this spectrum determined by their unique combination of genetic, environmental, and lifestyle factors [14]. Individuals classified as having “low cardiovascular risk” (LCR) based on traditional risk assessment tools may represent an essential population for studying the earliest stages of this continuum. While these individuals have a relatively low probability of experiencing cardiovascular events in the short term, they may perform subtle pathophysiological changes that could eventually progress to clinical disease [15].

We hypothesize that distinct alterations in the gut microbiome may be detectable even in individuals classified as having low cardiovascular risk when compared to healthy controls with no risk factors. These microbial changes might represent early signatures associated with the initial stages of CVD development, potentially preceding changes in traditional clinical parameters. To investigate this hypothesis, we performed shotgun metagenomic sequencing, aiming to identify specific microbial taxa and functional pathways that differ between low-risk individuals and healthy controls, to provide insight into the early microbiome shifts associated with CVD development. Understanding these changes could help improve risk assessment and preventive strategies targeting the gut microbiome to mitigate overall CVD risk prior to clinical manifestation.

## 2. Materials and Methods

### 2.1. Study Population

**Figure 1:**
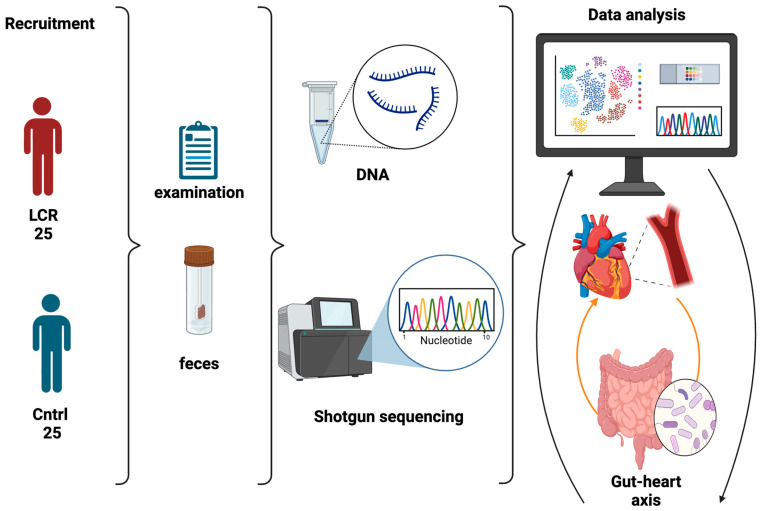
Study design. Note: Created in BioRender. Issilbayeva, A. (2025) https://BioRender.com/auskmo4, accessed on 23 April 2025, agreement No. UH286KCGZ3; 49 Spadina Ave. Suite 200 Toronto ON M5V 2J1 Canada www.biorender.com.

Our research involved 50 participants and included two groups: 25 individuals with low cardiovascular risk (LCR) and 25 age- and sex-matched healthy controls (Cntrl) (Figure 1).

Recruitment took place at the “Heart Center” of the University Medical Center in Astana (Nazarbayev University), Kazakhstan, between March and December 2024. Participants in the LCR group were identified through cardiovascular screening programs, with inclusion criteria requiring participants to be older than 18 and willing to complete all study requirements. The Control group consisted of volunteers who had no identifiable cardiovascular risk factors or disease upon thorough evaluation and no hereditary cardiac disease.

We applied several exclusion criteria across both study groups: use of medications that might affect gut microbiota (antibiotics, probiotics, antivirals) within 3 months before enrollment; the presence of inflammatory or autoimmune disorders; significant chronic illnesses; metabolic conditions; compromised immune conditions; gastrointestinal surgery or infection within six months; pregnancy; lactation; or inability to provide informed consent.

For cardiovascular risk classification, the European SCORE2 system was employed, which defines low risk as <2.5% for individuals under 50 years, <5% for those aged 50–65 years, and <7.5% for those 70 years and older, and in accordance with Appendix 3 of Clinical Protocol for Diagnosis and Treatment “Atherogenic Disorders of Lipid Metabolism (Dyslipidemia)” [16]. All participants underwent comprehensive laboratory evaluation, including hematological assessment, hepatic function tests, hemostatic and glycemic parameters, and detailed lipid profiling (TC, LDL-C, HDL-C, Non-HDL-C, TG, ApoA, ApoB, Lp(a)).

Study participants underwent additional cardiovascular evaluations, including 24 h ambulatory blood pressure monitoring (ABPM), standard 12-lead electrocardiogram (ECG), echocardiography, carotid ultrasound imaging, and peripheral arterial assessment to confirm the absence of subclinical atherosclerotic disease.

The Control group included individuals with no identifiable cardiovascular risk factors. Exclusion criteria for the Control group included any history of cardiovascular disease, diabetes mellitus, hypertension, dyslipidemia, smoking (current or past), obesity (BMI > 30 kg/m^2^), family history of premature cardiovascular events, or abnormal findings on physical examination, and abnormal laboratory and instrumental diagnostic parameters.

### 2.2. Biological Sample Collection

Fecal samples were collected within 48 h of study enrollment using Zymo Research DNA/RNA Shield™ Collection Tubes, with detailed instructions provided to participants. All samples were transported to the Microbiome Laboratory within 24 h and cryopreserved at −80 °C for subsequent analysis.

### 2.3. Microbiome Characterization

#### 2.3.1. Sequencing

We performed shotgun metagenomic sequencing to obtain a comprehensive profile of the gut microbiome composition. Bacterial DNA was isolated from fecal samples using the ZymoBIOMICS DNA Miniprep Kit Cat# D4301, Irvine, USA according to the manufacturer's standard protocol. Quality assurance involved multiple complementary methods: NanoDrop 2000 spectrophotometry, Qubit 3.0 fluorometric quantification, and agarose gel electrophoresis to confirm DNA integrity.

The sequencing libraries were prepared following established metagenomic protocols. Sequencing was performed on an Illumina NovaSeq 6000 system with paired-end read generation and a minimum sequencing depth of 6 Gb per sample to ensure adequate coverage of microbial genetic material.

#### 2.3.2. Computational Analysis Pipeline

Sequencing data underwent a multi-step bioinformatic processing. Initial quality control and preprocessing removed adapter sequences and low-quality reads using Trimmomatic (v0.39). For taxonomic classification, we employed MetaPhlAn 4, while functional profiling was conducted using HUMAnN 3 with the UniRef90 reference database to identify metabolic pathways.

#### 2.3.3. Data Analysis

Alpha diversity was evaluated using Observed and Shannon diversity indexes at the species genome bins (SGBs) level. Beta diversity was assessed using Jaccard and Bray–Curtis distances. ANOSIM and PERMANOVA tests with 999 permutations were used to evaluate the significance of grouping. For the PERMANOVA test, homogeneity of multivariate variances was examined using PERMDISP test; variances were considered comparable at *p* > 0.05. Beta diversity was visualized using a principal coordinate analysis (PCoA). To identify differentially abundant microbial taxa, we used Linear Discriminant Analysis with Effect Size calculation (LEfSe). The LEfSe LDA threshold was set to LDA ≥ 2 and *p* ≤ 0.05. Functional pathways were considered differentially abundant with non-overlapping 95% confidence intervals (CIs) for differences in means and *p* ≤ 0.05. Only features with at least 80% prevalence in at least one group were considered for the differential analysis. All pairwise comparisons were performed using an independent *T*-test with or without the Welch correction, or a Mann–Whitney U-test, depending on the data distribution. Counts comparison between groups was conducted using Fisher’s exact test. Associations between microbial features and cardiovascular parameters were examined using a generalized linear model (GLM), adjusting for age, BMI, and sex. Correlation between taxonomic and functional markers was assessed using Spearman correlation coefficient. Significance was established at *p* ≤ 0.05. All statistical analyses and visualizations were generated using Python v3.12, NumPy v2.0.1, SciPy v1.15.1, scikit-bio v0.6.3, Matplotlib v3.10.0, and seaborn v0.13.2 packages.

## 3. Results

The present study analyzed data from 25 participants in the LCR group and 25 participants in the Control group.

### 3.1. Analysis of Clinical-Demographic Data

The demographic and clinical characteristics of the LCR and control groups are summarized in Table 1. Both groups were comparable in terms of age (Control: 49.0 [46.0–55.0] vs. LCR: 50.0 [43.0–54.0] years, *p* = 0.41), sex distribution (68% female in both groups, *p* = 1.0), BMI (Control: 26.6 [23.9–28.1] vs. LCR: 25.3 [23.4–28.2] kg/m^2^, *p* = 0.89). There were no statistical differences in CRP, HDL, TCH, TG, ApoA, Lp(a), smoking, and alcohol consumption (*p* > 0.05), Table 1.

However, significant differences were observed in HbA1c levels, which were higher in the Control group compared to the LCR group (5.6 [5.4–5.9] vs. 5.2 [4.6–5.6]%, *p* < 0.01), while LDL cholesterol (Control: 109.5 [91.3–125.4] vs. LCR: 123.6 [109.3–138.3] mg/dL, *p* < 0.01) and ApoB (Control: 0.8 [0.7–0.9] vs. LCR: 0.9 [0.8–1.0] mg/dL, *p* < 0.05) were significantly higher in the LCR group.

A descriptive analysis of the main study group with LCR demonstrated that a family history of cardiovascular disease was reported by 52.0% of participants. Most participants did not have a family history of diabetes (92.0%) or first-degree relatives with CAD/CVD/LDL disorders (92.0%). A family history of cancer was reported by 24.0% of participants. Notably, none of the participants had a personal history of early coronary artery disease (CAD). All participants (100%) were free of diabetes mellitus, while 4.0% had impaired glucose tolerance. The median fasting glucose was 102.4 mg/dL [IQR: 94.0–104.9], and the median HbA1c was 5.2% [IQR: 4.6–5.6].

None of the participants had a history of myocardial infarction, stroke, or revascularization procedures. Hypertension was absent in 72.0% of the participants, while 16.0% had Stage 1 hypertension and 12.0% had Stage 2 hypertension. All participants had normal ECG findings with sinus rhythm (100.0%). The median ejection fraction was 63.0% [IQR: 61.9–65.0], and the median global longitudinal strain was −24.7% [IQR: −25.9–−22.8]. Aortic valve assessment showed no changes in 82.6% of participants, while 17.4% had thickened cusps. None of the participants exhibited left ventricular hypertrophy. Coronary artery calcium (CAC) scoring showed no calcification in 91.7% of participants, with only 8.3% showing moderately high calcium scores. All participants had normal findings on ultrasonography of peripheral arteries (100%).

The median neutrophil-to-lymphocyte ratio was 1.5 [IQR: 1.2–2.1]. Platelets were within the normal range at 265.0 × 10^9^/L [IQR: 224.0–293.0], and fibrinogen was 2.8 g/L [IQR: 2.4–3.2]. The median creatinine level was 0.8 mg/dL [IQR: 0.7–0.9], with a median estimated glomerular filtration rate (GFR) of 100.1 mL/min/1.73 m^2^ [IQR: 89.2–111.5].

### 3.2. Microbiome Analysis

#### 3.2.1. Compositional Characteristics of Gut Microbiome

**Figure 2:**
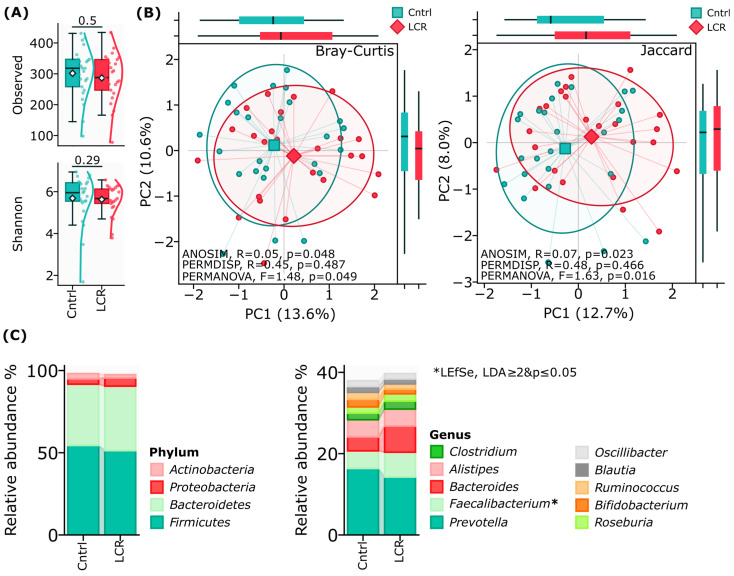
Biodiversity analysis of gut microbiome composition between control (Cntrl) and low cardiovascular risk (LCR) patients. (**A**) Within-sample diversity measured by Observed and Shannon indices. (**B**) Principal coordinate analysis (PCoA) of between-sample diversity was assessed using Bray–Curtis and Jaccard metrics. ANOSIM, PERMANOVA grouping tests with 999 permutations. (**C**) Stackplot of relative taxa abundances at the phylum and genus level. “*” indicates significant change as determined by LEfSe, LDA ≥ 2 and *p* ≤ 0.05.

Alpha diversity metrics revealed no significant differences between the LCR and Control groups. There was similar taxonomic richness between the study groups according to Observed (*p* > 0.05) and Shannon diversity index (*p* > 0.05), Figure 2A.

Analysis of beta diversity revealed distinct microbial community structures between LCR and Control groups based on Bray–Curtis dissimilarity metrics, confirming separation between the microbial communities, with 24.2% of the total variation and moderate effect sizes (ANOSIM, R = 0.05 (*p* = 0.048), while PERMANOVA, F = 1.48 (*p* = 0.049)), and Jaccard dissimilarity metrics, with 20.7% of total variation (ANOSIM, R = 0.07 (*p* = 0.023), while PERMANOVA, F = 1.63 (*p* = 0.016)), Figure 2B, Supplementary Table S1.

The relative abundance analysis revealed a predominance of *Firmicutes* in both the control and LCR groups (54.5 ± 19.5% vs. 51.3 ± 16.8%), followed by *Bacteroidetes* (37.2 ± 20.5% vs. 39.2 ± 18.5%), with no significant differences at the phylum level. At the class level, considerable enrichment of *Gammaproteobacteria* (0.8 ± 1.0% vs. 1.4 ± 5.4%, *p* = 0.04) and a substantial decrease in *Erysipelotrichia* (0.7 ± 0.7% vs. 0.2 ± 0.3%, *p* = 0.0001) were observed in the LCR group. The order-level distribution showed consistent patterns of increased *Enterobacterales* (0.7 ± 0.0% vs. 1.4 ± 5.4%, *p* = 0.03) and decreased *Erysipelotrichales* (0.7 ± 0.7% vs. 0.2 ± 0.3%, *p* = 0.0001) in the LCR group. At the family level, among the top ten taxa, *Enterobacteriaceae* showed significant enrichment in LCR samples (0.7 ± 0.9% vs. 1.4 ± 5.4%, *p* = 0.03). The genus-level analysis identified a substantial increase in *Faecalibacterium* (4.3 ± 3.1% vs. 6.1 ± 3.3%, *p* = 0.027) in the LCR group, while at the species level, *Faecalibacterium prausnitzii* (4.0 ± 2.9% vs. 5.6 ± 3.1%, *p* = 0.035) represented the most prevalent taxa, showing significant elevation in the LCR group compared to controls, Figure 2C, Supplementary Figure S1, Table S2.

LEfSe analysis identified multiple bacterial taxa significantly differentiating between LCR and Control groups (LDA > 2, *p* ≤ 0.05). The Control group demonstrated enrichment in *Tannerellaceae*, *Erysipelotrichaceae*, *Enterobacteriaceae* families and the *Senegalimassilia*, *Catenibacterium,* and *Gammaproteobacteria* genera. Conversely, the LCR group exhibited a significantly higher abundance of multiple taxa within the *Eggerthellaceae* family, and *Clostridium*, *Lachnospira*, *Dysosmobacter*, *Anaerotruncus*, *Faecalibacterium*, *Bilophila*, and *Firmicutes* genera.

**Figure 3:**
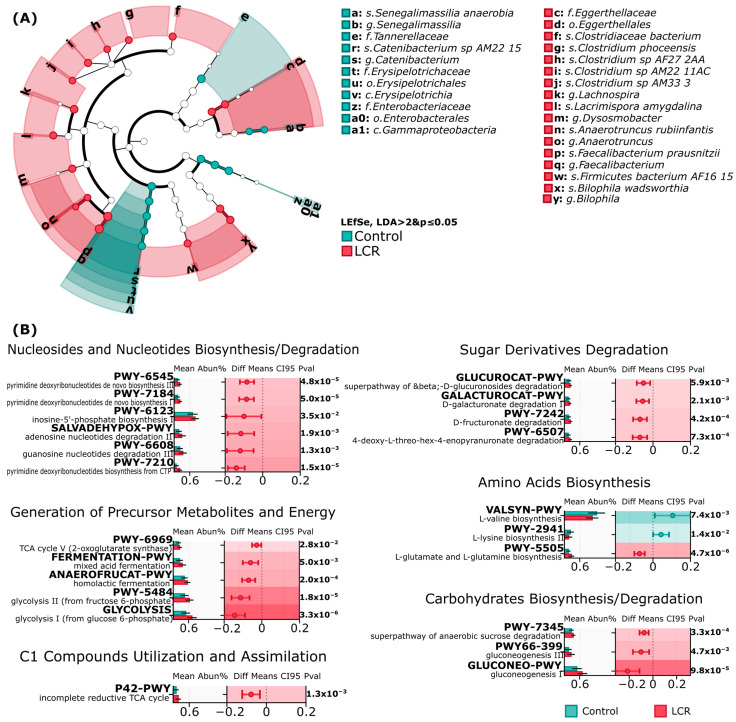
Taxonomic and functional markers of Control (Cntrl) and low cardiovascular risk (LCR) patients’ microbiomes. (**A**) LEfSe cladogram indicating differentially abundant taxa. LEfSe, LDA ≥ 2 and *p* ≤ 0.05. (**B**) Confidence plots of differentially abundant inferred MetaCyc pathways grouped by functional modules. Non-overlapping 95%CI and *p* ≤ 0.05.

Notably, taxa with the highest LDA scores included *s. Faecalibacterium prausnitzii* (LDA = 3.18, *p* = 0.03) in LCR and *s. Catenibacterium*_sp_AM22_15 (3.496), (LDA = 3.10, *p* < 0.0001) in the Control groups, indicating their significant differential abundance. Additionally, LCR samples showed increased abundance of *Erysipelotrichaceae* family members, *Anaerotruncus rubiinfantis*, and the sulfite-reducing bacterium *Bilophila wadsworthia*, Figure 3A,B, Supplementary Figure S2. The rest of the LDA scores and *p*-values are available in Supplementary Table S3.

#### 3.2.2. Pathway Analysis of Gut Microbiome

The analysis of metabolic pathways between the Control and LCR groups revealed upregulation of core metabolic functions in the LCR group compared to the Control, Figure 3B, suggesting impaired energy production, nucleotide synthesis, and carbohydrate metabolism. The nucleosides and nucleotides biosynthesis/degradation pathways showed significant increases in the LCR group, particularly for pyrimidine deoxyribonucleotides de novo biosynthesis III (PWY-6545, *p* = 4.8 × 10^−5^, |Δmean| = 0.1), pyrimidine deoxyribonucleotides de novo biosynthesis I (PWY-7184, *p* = 5.0 × 10^−5^, |Δmean| = 0.1), and pyrimidine deoxyribonucleotides biosynthesis from CTP (PWY-7210, *p* = 1.5 × 10^−5^, |Δmean| = 0.1), inosine-5′-phosphate biosynthesis I (PWY-6123, *p* = 0.035, |Δmean| = 0.1), adenosine nucleotides degradation II (SALVADEHYPOX-PWY, *p* = 0.002, |Δmean| = 0.1), and guanosine nucleotides degradation III (PWY-6608, *p* = 1.3 × 10^−3^, |Δmean| = 0.1).

In the generation of precursor metabolites and energy category, glycolysis pathways were comparably increased in LCR (GLYCOLYSIS, *p* = 3.3 × 10^−6^; PWY-5484, *p* = 1.8 × 10^−5^, |Δmean| = 0.2), as were fermentation pathways (FERMENTATION-PWY, *p* = 5.0 × 10^−3^, |Δmean| = 0.1; ANAEROFRUCAT-PWY, *p* = 2.0 × 10^−4^, |Δmean| = 0.1) and TCA cycle V (2-oxoglutarate synthase) (PWY-6969, *p* = 2.8 × 10^−2^, |Δmean| = 0.1).

Sugar derivative degradation showed consistent increases in the LCR group across all pathways, including D-glucuronosides (GLUCUROCAT-PWY, *p* = 5.9 × 10^−3^ б |Δmean| = 0.1), D-galacturonat (GALACTUROCAT-PWY, *p* = 2.1 × 10^−3^, |Δmean| = 0.1), 4-deoxy-L-threo-hex-4-enopyranuronate (PWY-6507, *p* = 7.3 × 10^−4^, |Δmean| = 0.1) and D-fructuronate degradation (PWY-7242, *p* = 4.2 × 10^−4^, |Δmean| = 0.1).

For amino acid biosynthesis, L-glutamate/glutamine biosynthesis increased in the LCR group (PWY-5505, *p* = 4.7 × 10^−6^, |Δmean| = 0.1), while L-valine (VALSYN-PWY, *p* = 7.4 × 10^−3^, |Δmean| = 0.2) and L-lysine (PWY-2941, *p* = 1.4 = 10^−2^, |Δmean| = 0.1) biosynthesis pathways, on the contrary, increased in the Control group.

Carbohydrate metabolism pathways increased in the LCR group, particularly gluconeogenesis (GLUCONEO-PWY, *p* = 9.8 × 10^−5^, |Δmean| = 0.2; PWY66-399, *p* = 4.7 × 10^−3^, |Δmean| = 0.1) and anaerobic sucrose degradation (PWY-7345, *p* = 3.3 × 10^−4^, |Δmean| = 0.1).

The C1 compound utilization and assimilation pathway (incomplete reductive TCA cycle) was also significantly increased in the LCR group (P42-PWY, *p* = 1.3 × 10^−3^, |Δmean| = 0.1), Figure 3B, Supplementary Table S4.

#### 3.2.3. Integrative Analysis of Gut Microbiome Composition

**Figure 4:**
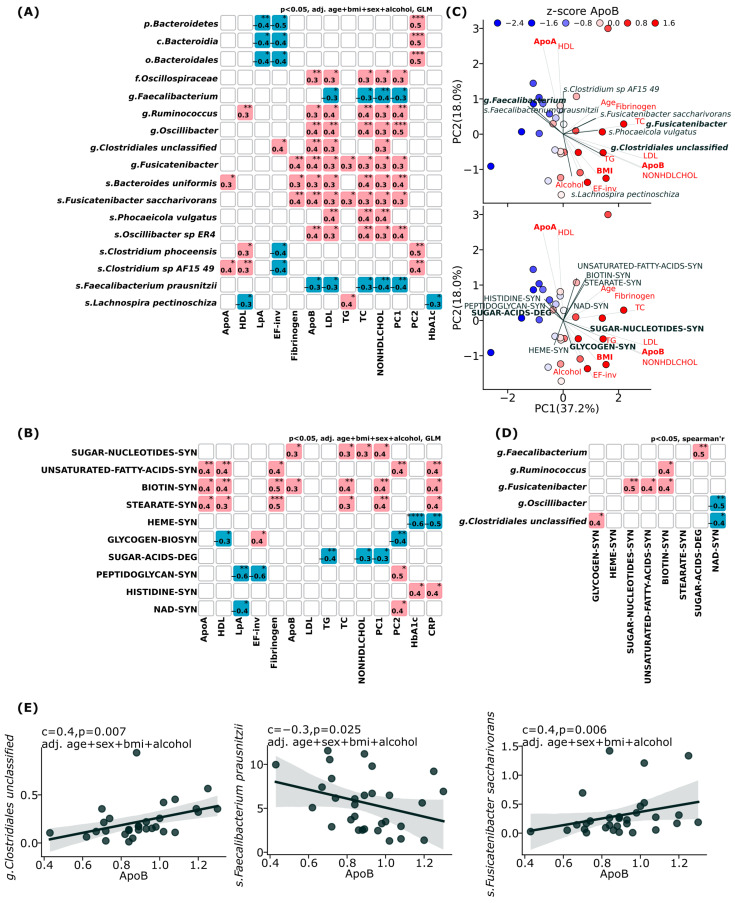
Association analysis between cardiovascular risk factors and gut microbiome features in low cardiovascular risk (LCR) patients. Principal coordinate analysis (PCA) decomposition of cardiovascular risk factors data and its association with identified taxonomic and functional markers. + as well as demographic parameters. (**A**) Significant associations between cardiovascular risk factors and gut microbiome taxa. Generalized linear model (GLM), *p* ≤ 0.05, adjusted for age, BMI, sex, and alcohol consumption. ‘c’—coefficient from GLM model. Only taxa with at least two significant associations with risk factors were considered significant. (**B**) Significant associations between cardiovascular risk factors and inferred gut microbiome functional features. Same method as in (**A**). (**C**) Principal coordinate analysis (PCA) decomposition of cardiovascular risk factors data and its association with identified taxonomic and functional markers. Scaling 3. (**D**) Correlation between identified taxonomic and functional markers. Spearman rho. ‘r’—correlation coefficient. (**E**) Scatterplots for three salient identified taxonomic markers and ApoB levels. ApoA = apolipoprotein A, HDL = high-density lipoprotein, LpA = lipoprotein A, EF-inv = inverted ejection fraction %, ApoB = apolipoprotein B, LDL = low-density lipoprotein, TG = triglycerides, TC = total cholesterol, NONHDLCHOL = total cholesterol minus high-density lipoprotein cholesterol, PC1/2 = first/second component of cardiovascular risk factors PCA decomposition, HbA1c = hemoglobin A1C, CRP = C-reactive protein, BMI = body mass index. * ≤0.05, ** ≤0.01, *** ≤0.001, **** ≤0.0001.

Our correlation analysis revealed significant associations between the gut microbiome composition and cardiovascular risk factors in the LCR group, Figure 4. After adjusting for potential confounders (age, BMI, sex, and alcohol consumption), we identified specific bacterial taxa with significant positive or negative correlations with atherogenic lipid markers, Figure 4A, Supplementary Table S5. Several genera, including *Oscillibacter*, *Ruminococcus*, *Fusicatenibacter*, and *unclassified Clostridiales*, showed significant positive correlations (r = 0.3–0.5, *p* ≤ 0.05) with atherogenic lipid markers such as ApoB, non-HDL cholesterol, triglycerides, and the first principal component (PC1). At the species level, *Bacteroides uniformis*, *Fusicalenibacter saccharivorans*, *Phocaeicola vulgatus*, and *Oscillibacter* sp. *ER4* similarly demonstrated positive correlations with these atherogenic markers. Conversely, the genus *Faecalibacterium* and its species *Faecalibacterium prausnitzii* exhibited significant negative correlations (r = −0.3 to −0.5, *p* ≤ 0.05) with the same atherogenic parameters. *Faecalibacterium prausnitzii* notably showed inverse relationships with ApoB, non-HDL cholesterol, and PC1.

Correlation analysis of metabolic pathways and lipid parameters revealed notable findings in SUGAR-NUCLEOTIDES synthesis pathways showing positive associations with ApoB, triglycerides, and PC1 (r = 0.3–0.4, *p* ≤ 0.05). Interestingly, pathways involved in unsaturated fatty acids biosynthesis, biotin synthesis, and stearate biosynthesis demonstrated negative correlations with ApoB and other atherogenic markers while positively correlating with HDL and ApoA, Figure 4B, Supplementary Table S5.

Further, PCA was performed to explore relationships between microbial taxa, metabolic pathways, and clinical parameters. The first two principal components explained 55.2% (37.2% and 18.0%, respectively) of the total variance. Apolipoprotein B distribution demonstrated a clear clustering pattern along the PC1 axis, with ApoB, Non-HDL- cholesterol and LDL demonstrating an inverse relationship with ApoA and HDL. The specific taxa vectors revealed a contrasting relationship between *Feacalibacterium prausnitzii* and *Fusicatanibacter saccharivorans* with lipid parameters, Figure 4C. The analysis also highlighted significant clustering of metabolic pathways, with notable contributions from sugar-nucleotide synthesis and sugar acid degradation pathways, Figure 4C, Supplementary Table S5.

Correlation analysis revealed significant associations between bacterial genera and metabolic pathways in the low cardiovascular risk (LCR) group (Figure 4D). Notably, the *Faecalibacterium* genus demonstrated a strong positive correlation with sugar acid degradation (r = 0.5, *p* = 0.01), suggesting its contribution to cellular energy production. *Fusicatenibacter* exhibited robust positive correlations with multiple anabolic pathways, including biotin synthesis (r = 0.4, *p* = 0.04), sugar-nucleotide synthesis (r = 0.5, *p* = 0.004), and unsaturated fatty acid biosynthesis (r = 0.4, *p* = 0.02), highlighting its potential role in carbohydrate and lipid metabolism regulation. *Ruminococcus* showed moderate positive associations with biotin synthesis (r = 0.4, *p* < 0.02), while unclassified *Clostridiales* (r = −0.5, *p* = 0.03) and *Oscillibacter* (r = −0.4, *p* = 0.007) negatively correlated with NAD synthesis. *unclassified Clostridiales* positively correlated with the glycogen biosynthesis pathway (r = 0.4, *p* = 0.02), Figure 4D, Supplementary Table S5.

The scatter plots quantify these relationships in Figure 4E, Supplementary Figure S3, demonstrating a negative correlation between genus *Faecalibacterium* abundance and ApoB levels (c = −0.3, *p* = 0.056), which becomes more significant at the species level for *Faecalibacterium prausnitzii* (c = −0.3, *p* = 0.025). Conversely, genus *Clostridiales unclassified* (c = 0.4, *p* = 0.007), *Fusicatenibacter* (c = 0.4, *p* = 0.006), and *Fusicatenibacter saccharivorans* (r = 0.4, *p* = 0.006) species exhibited a significant positive correlation with ApoB. These relationships persisted after adjusting for age, sex, BMI, and alcohol consumption, Figure 4E, Supplementary Table S5. Among other taxa, the *Oscillibacter* genus demonstrated positive correlations with ApoB (c = 0.4, *p* = 0.008), with *Oscillibacter* species ER4 showing a similar association pattern (c = 0.4, *p* = 0.007). *Ruminococcus* exhibited a moderate positive correlation with ApoB (c = 0.3, *p* = 0.024), while *Bacteroides uniformis* (panel I) also shows a positive association (c = 0.3, *p* = 0.016), Supplementary Figure S3, Table S5.

## 4. Discussion

The gut microbiome and its metabolic pathways play a significant role in the pathogenesis and prevention of cardiovascular disease [17]. Our study provides insights into the gut microbiome composition of individuals with low cardiovascular risk (LCR), revealing distinct microbial signatures that may represent early changes associated with cardiovascular disease development. While alpha diversity metrics showed no significant differences between LCR and Control groups, beta diversity analysis demonstrated clear separation of microbial communities, suggesting subtle yet meaningful alterations in microbial composition even before the manifestation of clinical cardiovascular disease.

The most notable finding in our study was the significant enrichment of *Faecalibacterium prausnitzii* in the LCR group. This pattern aligns with the evidence summarized by Leylabadlo et al., who documented increased *Faecalibacterium prausnitzii* abundance across numerous inflammatory and metabolic conditions, including type 2 diabetes, inflammatory bowel diseases, and Crohn’s disease [18]. This commensal bacterium has been extensively studied for its anti-inflammatory properties [19,20,21] and potential cardioprotective effects [22,23], producing several bioactive compounds, including butyrate, shikimic acid, and salicylic acid, which collectively contribute to maintaining intestinal homeostasis and reducing systemic inflammation [24]. The dominance of this bacterium in early disease stages likely represents an attempted compensatory mechanism by the gut microbiome to counteract developing inflammatory processes. Studies have linked its abundance to a lower incidence of coronary artery disease and demonstrated its ability to mitigate atherosclerosis in animal models [25]. In recent human studies, lifestyle-based interventions have increased the abundance of these bacteria and improved cardiovascular health markers [26]. Martin et al. reported that *Faecalibacterium prausnitzii* mitigated physiological damage in a mouse model of chronic low-grade inflammation. The study demonstrated that this beneficial gut bacterium plays a protective role against inflammatory processes, suggesting its potential therapeutic value in managing inflammatory conditions [27]. Notably, *Faecalibacterium prausnitzii* has been previously demonstrated to inhibit atherosclerotic deposit formation and lower lipid levels in a mouse model [28]. These experimental findings align with our observational data, which revealed significant negative correlations between *Faecalibacterium prausnitzii* abundance and adverse lipid parameters, including ApoB. Existing data align with our results, which demonstrated that this beneficial bacterium dominated in subjects with low cardiovascular risk, suggesting a compensatory protective mechanism during the early stages of disease development. We can hypothesize that the abundance of *Faecalibacterium prausnitzii* appears to serve as a natural defensive response, potentially counteracting inflammatory processes before they progress to more severe cardiovascular complications. The observed changes are associative and not causal, and further longitudinal studies are essential.

Another notable finding was a taxon with opposite patterns to *Faecalibacterium prausnitzii* in the LCR group. *Fusicatenibacter saccharivorans*, a member of the *Lachnospiraceae* family, is a gram-positive, anaerobic bacterium found in the human intestine [29]. Studies have shown that *F. saccharivorans* decreases in active ulcerative colitis (UC) patients and increases during remission. The administration of *F. saccharivorans* improved murine colitis and induced interleukin-10 production in lamina propria mononuclear cells in both colitis model mice and UC patients, suggesting its potential as a novel UC treatment [30]. While *Fusicatenibacter saccharivorans* shows promise in treating inflammatory bowel disease, in our study, *Fusicatenibacter saccharivorans* (r = 0.4, *p* = 0.006) exhibited a significant positive correlation with ApoB. This finding presents an interesting contrast with its previously reported anti-inflammatory properties. This parallel observation strengthens the emerging evidence that *Fusicatenibacter saccharivorans* may serve as a potential microbial marker in the liver-gut microbiota-heart axis, with its unique relationship to lipoprotein metabolism distinguishing it from other gut bacteria involved in cardiovascular health. Our results partly correspond with the findings from Hu et al., who observed an increase in this taxon in coronary artery disease complicated with nonalcoholic fatty liver disease patients (CAD-NAFLD) [31]. The positive correlation of this taxon with ApoB, a key component of atherogenic lipoproteins and a well-established cardiovascular risk marker, suggests that *Fusicatenibacter saccharivorans* may play a more complex role in cardiometabolic health than previously understood. As shown in our correlation heatmap, *Fusicatenibacter saccharivorans* also demonstrated positive associations with fibrinogen, ApoB, LDL, TC, and various principal components related to lipid metabolism. These correlations suggest that *F. saccharivorans* shows unfavorable relationships with lipid metabolism and cardiovascular risk factors. This contrasting pattern with *Fusicatenibacter saccharivorans*, which dominated in subjects with low cardiovascular risk, highlights the complex interplay between different gut microbiome members and their potentially opposing roles in cardiovascular health.

Our findings reveal significant differences in multiple metabolic pathways between individuals with LCR and Control subjects. These alterations primarily affect nucleoside/nucleotide metabolism, energy generation, sugar derivative degradation, amino acid biosynthesis, and carbohydrate metabolism, suggesting altered metabolic activity in the LCR phenotype.

The upregulation of nucleoside and nucleotide biosynthesis/degradation pathways in LCR individuals, particularly the pyrimidine deoxyribonucleotides de novo biosynthesis (PWY-6545, PWY-7184), suggests enhanced cellular renewal capacity. These findings correspond to the existing data that pyridine nucleotides NAD(+)/NADH and NADP(+)/NADPH regulate intermediary metabolism, energy substrate preference, and oxidative stress in the heart [32]. Purine nucleotides, particularly ATP, are essential for cardiac energetics and mechanics. Perturbations to purine nucleotide metabolism in the heart can lead to impaired myocardial energetics and mechanics in cardiac disease; precisely, depletion of adenine nucleotides is associated with impaired myocardial function in acute ischemia and heart failure [33,34].

Our findings reveal significant differences in amino acid biosynthesis pathways between individuals with LCR and control subjects. Notably, amino acid biosynthesis pathways, including L-valine (VALSYN-PWY, *p* = 7.4 × 10^−3^), L-lysine (PWY-2941, *p* = 1.4 × 10^−2^), show reduced activity in the LCR group. This is particularly relevant given recent research by Xiong et al. [35] and Gao et al. [36] linking altered branched-chain amino acid (BCAA) metabolism to various cardiovascular conditions. The observed downregulation of valine biosynthesis in our LCR cohort may represent a protective metabolic adaptation that prevents harmful BCAA accumulation associated with insulin resistance and cardiometabolic diseases [37]. This metabolic signature aligns with Huang et al.’s findings on the importance of balanced amino acid metabolism in cardiac health [38].

Our findings demonstrate significant upregulation of energy generation pathways in individuals with low cardiovascular risk (LCR) compared to controls. Notably, glycolytic pathways show substantial enhancement in the LCR group, with glycolysis I (from glucose 6-phosphate) displaying the most pronounced difference (*p* = 3.3 × 10^−6^), followed by glycolysis II (from fructose 6-phosphate) (*p* ≤ 0.0001 This increased glycolytic capacity aligns with Newhardt et al.’s observation that enhanced glycolysis provides cardioprotection against diet-induced cardiomyopathy [39]. Additionally, the LCR group exhibits significantly elevated TCA cycle activity (PWY-6969, *p* ≤ 0.05) and fermentation pathways (FERMENTATION-PWY, *p* ≤ 0.05), ANAEROFRUCAT-PWY, *p* ≤ 0.001 suggesting a comprehensive metabolic adaptation that promotes energetic flexibility. These findings contradict the view that shifts from fatty acid oxidation to glycolysis contribute to cardiac pathogenesis [40]. Instead, our results suggest that enhanced glycolytic capacity may represent a beneficial metabolic adaptation in cardiovascular health, potentially providing greater energetic reserve during metabolic stress. This supports Chen et al.’s proposition that glycolytic enzymes could serve as therapeutic targets, though our data suggests the goal might be pathway enhancement rather than inhibition in specific clinical contexts [41]. The observed metabolic signature in our LCR cohort provides new insights into how modulation of energy metabolism pathways, as suggested by Zuurber et al. and Lopaschuk et al., might be a key point in cardiovascular risk [42,43].

The LCR group also exhibited markedly elevated gluconeogenesis (GLUCONEO-PWY, *p* ≤ 0.001) and anaerobic sucrose degradation (PWY-7345, *p* ≤ 0.001) compared to Controls. This metabolic signature aligns with observations regarding the importance of glucose utilization in cardiac health, though through mechanisms different from those observed in heart failure [44].

Interestingly, our correlation and PCA analysis revealed notable opposite contributions from sugar-nucleotide synthesis and sugar acid degradation pathways corresponding to the recent review by Roessler et al. declaring that the microbiome can modulate cholesterol and glucose metabolism, impacting cardiovascular disease risk [45].

## 5. Conclusions

Our study provides novel insights into the gut microbiome composition of individuals with low cardiovascular risk, revealing distinct microbial signatures that may represent early alterations associated with cardiovascular disease development. Despite similar alpha diversity metrics, significant differences in community structure were identified between LCR and healthy control groups. The enrichment of genus *Faecalibacterium* and species *Faecalibacterium prausnitzii* and its negative correlation with atherogenic lipid markers suggest potential protective effects, while positive associations between genus *Fusicatenibacter* and *Fusicatenibacter saccharivorans* with unfavorable lipid profiles indicate their possible contribution to cardiometabolic risk. Specific bacterial taxa demonstrated potential relationships with key metabolic pathways, highlighting the functional consequences of taxonomic shifts. These findings suggest that gut microbiome signatures may serve as sensitive indicators of early metabolic dysregulation preceding clinically significant cardiovascular disease. Further longitudinal and interventional studies are required to determine causality and explore potential microbiome-targeted preventive strategies for cardiovascular health.

## 6. Limitations

Our study has several limitations that should be acknowledged. First, the cross-sectional design does not provide the determination of causal relationships between gut microbiome alterations and cardiovascular risk. Second, despite careful matching, our relatively small sample size (25 participants per group) may have limited statistical power to detect more subtle differences in microbial composition or metabolic pathways. Third, while we controlled for several confounding factors (age, BMI, sex, alcohol consumption, etc.), additional parameters which could influence both microbiome composition and cardiovascular parameters should be collected in future studies. Fourth, shotgun metagenomic sequencing, though comprehensive, may not fully capture the functional activities of the microbiome; complementary metabolomic and transcriptomic approaches would provide more robust evidence of bacterial functionality. Finally, the absence of validation in an independent cohort necessitates a cautious interpretation of our findings until they can be replicated in larger, more diverse populations. Future longitudinal studies with multi-omic approaches are needed to address these limitations and fortify our findings of gut microbiome signatures in early cardiovascular risk assessment.

## Figures and Tables

**Table 1:** Demographic and clinical characteristics of the study participants.

Parameter	Cntrl, n = 25	LCR, n = 25	*p*-Value
**Age, yrs** ^1^	49 [46.0–55.0]	50 [43.0–54.0]	0.414 ^a^
**Sex, f:m (%)**	17:8 (68:32)	17:8 (68:32)	1.0 ^b^
**BMI, kg/m^2^** ^1^	26.6 [23.9–28.1]	25.3 [23.4–28.2]	0.892 ^a^
**Smoking, n (%)**	0 (0%)	1 (4%)	1.0 ^b^
**Alcohol, n (%)**	9 (36%)	10 (40%)	1.0 ^b^
**HbA1c, %** ^1^	5.6 [5.4–5.9]	5.2 [4.6–5.6]	**0.004** ^c^
**CRP, mg/L** ^1^	0.1 [0.1–0.2]	0.1 [0.0–0.2]	0.540 ^a^
**HDL, mg/dL** ^1^	57.3 [50.3–62.3]	57.0 [53.6–67.8]	0.344 ^c^
**LDL, mg/dL** ^1^	109.5 [91.3–125.4]	123.6 [109.3–138.3]	**0.007** ^a^
**TG, mg/dL** ^1^	90.1 [62.0–124.8]	90.2 [52.8–132.0]	0.834 ^a^
**TCH, mg/dL** ^1^	189.6 [173.4–196.2]	187.7 [176.1–212.6]	0.230 ^a^
**Lp(a), mg/dL** ^1^	7.5 [5.9–16.8]	9.8 [7.6–22.4]	0.076 ^a^
**ApoA, g/L** ^1^	1.4 [1.2–1.5]	1.4 [1.3–1.6]	0.218 ^a^
**ApoB, g/L** ^1^	0.8 [0.7–0.9]	0.9 [0.8–1.0]	**0.020** ^a^

^1^—Md[IQR]. ^a^—Mann-Whitney U rank test; ^b^—Fisher’s exact test; ^c^—Ind. *T*-test. Bold values indicate statistically significant differences (*p* < 0.05) between Control and LCR groups.

## Data Availability

Raw sequencing data from this study have been deposited in the National Center for Biotechnology Information (NCBI) Sequence Read Archive under accession number PRJNA1255807.

## Supplementary Materials

The following supporting information can be downloaded at: https://www.mdpi.com/article/10.3390/jcm14145097/s1, Figure S1: Stackplot of relative taxa abundances. “*” indicates significant change as determined by LEfSe, LDA ≥ 2 and *p* ≤ 0.05. (A) At the Phylum level, (B) Class, (C) Order, (D) Family, (E) Genus, (F) Species; Figure S2: LEfSe barplot indicating differentially abundant taxa between control (Cntrl) and low cardiovascular risk (LCR) patients' microbiomes. LEfSe, LDA ≥ 2 and *p* ≤ 0.05. (A) At the Class level, (B) Order, (C) Family, (D) Genus, (E) Species; Figure S3: Scatterplots for identified taxonomic markers and ApoB levels. Generalized linear model (GLM), *p* ≤ 0.05, adjusted for age, BMI, sex, and alcohol consumption. (A) Association between ApoB levels and family *Oscillospiraceae* abundance, (B) genus *Fusicatenibacter*, (C) unclassified genus *Clostridiales*, (D) genus *Oscillibacter*, (E) genus *Ruminococcus*, (F) genus *Faecalibacterium*, (G) species *Fusicatenibacter saccharivorans*, (H) species *Oscillibacter ER4*, (I) species *Bacteroides uniformis*, (J) species *Faecalibacterium prausnitzii*; Table S1: Additional data to Figure 1; Table S2: Additional data to Supplementary Figure S1; Table S3: Additional data to Figure 3A; Table S4: Additional data to Figure 3B; Table S5: Additional data to Figure 4: A: Association analysis between cardiovascular risk factors and gut microbiome features in low cardiovascular risk (LCR) patients; B: Association analysis between cardiovascular risk factors and gut microbiome pathways in low cardiovascular risk (LCR) patients; C: PCA decomposition of cardiovascular risk factors; D: Correlation analysis between taxonomic and functional profiles; E: Scatterplots of taxonomic markers.

## Abbreviations

The following abbreviations are used in this manuscript:

CVD	Cardiovascular diseases
LCR	Low cardiovascular risk
Cntrl	Control group
TCH	Total cholesterol
LDL-C	Low-Density Lipoprotein Cholesterol
HDL-C	High-Density Lipoprotein Cholesterol
Non-HDL-C	Non-High-Density Lipoprotein Cholesterol
TG	Triglycerides
ApoA	Apolipoprotein A
ApoB	Apolipoprotein B
SCORE2	Systematic Coronary Risk Evaluation 2
ABPM	Ambulatory blood pressure monitoring
LEfSe	Linear Discriminant Analysis Effect Size
LDA	Linear Discriminant Analysis
MetaPhlAn	Metagenomic Phylogenetic Analysis
HUMAnN	Human Microbiome Project Unified Metabolic Analysis Network
UniRef90	Universal Protein Resource Reference Clusters 90%
BMI	Body Mass Index
EF	Ejection fraction
