# Supplementary figures and images for: Metagenomic Characterization of Gut Microbiota in Individuals with Low Cardiovascular Risk
Source: J Clin Med. 2025 Jul 17;14(14):5097. doi: 10.3390/jcm14145097 (PMC12295110; doi:10.3390/jcm14145097)

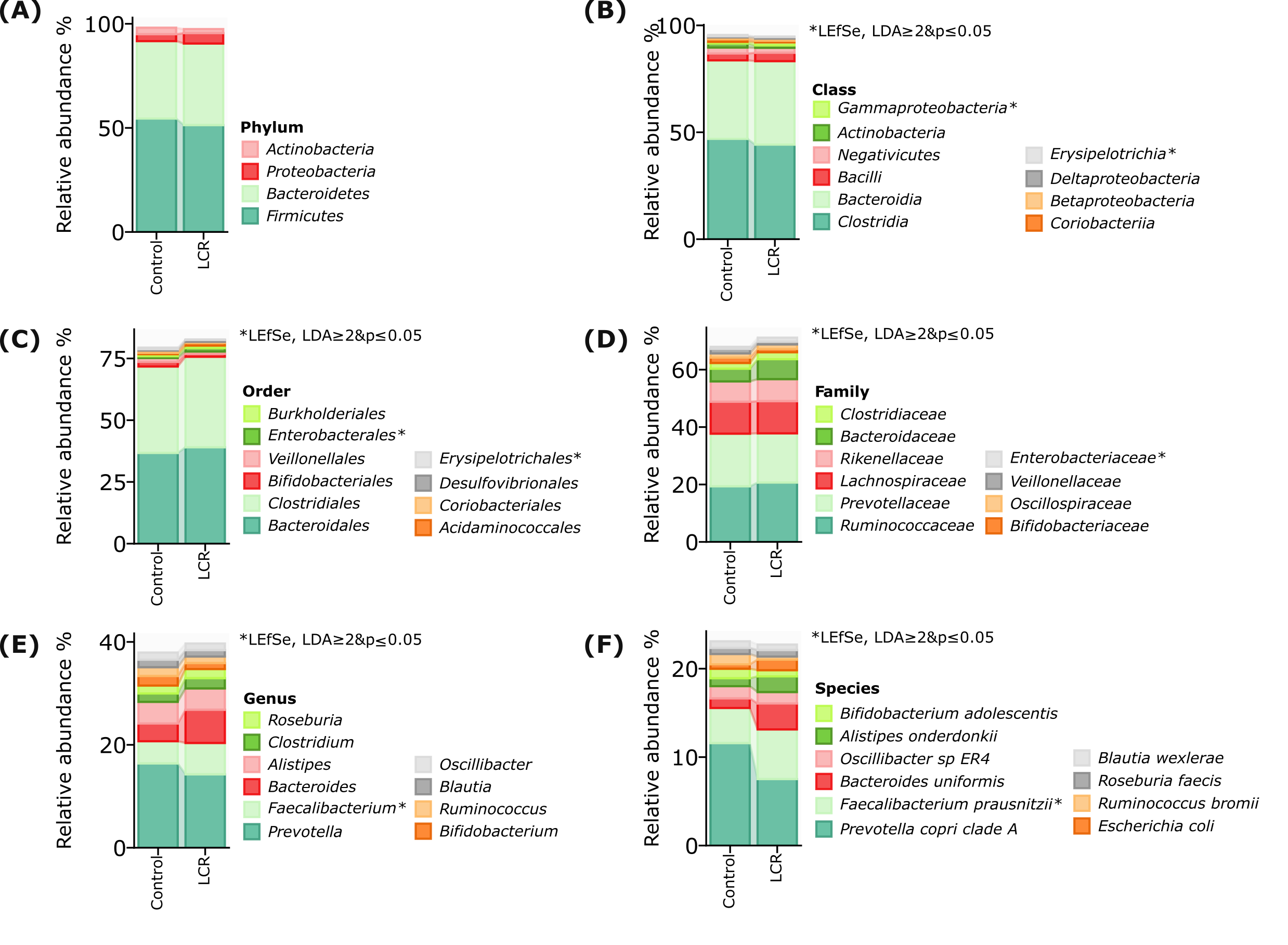

Supplement: Supplementary file 1 [file jcm-14-05097-s001.zip › Suppl figures/Figure S1.png]

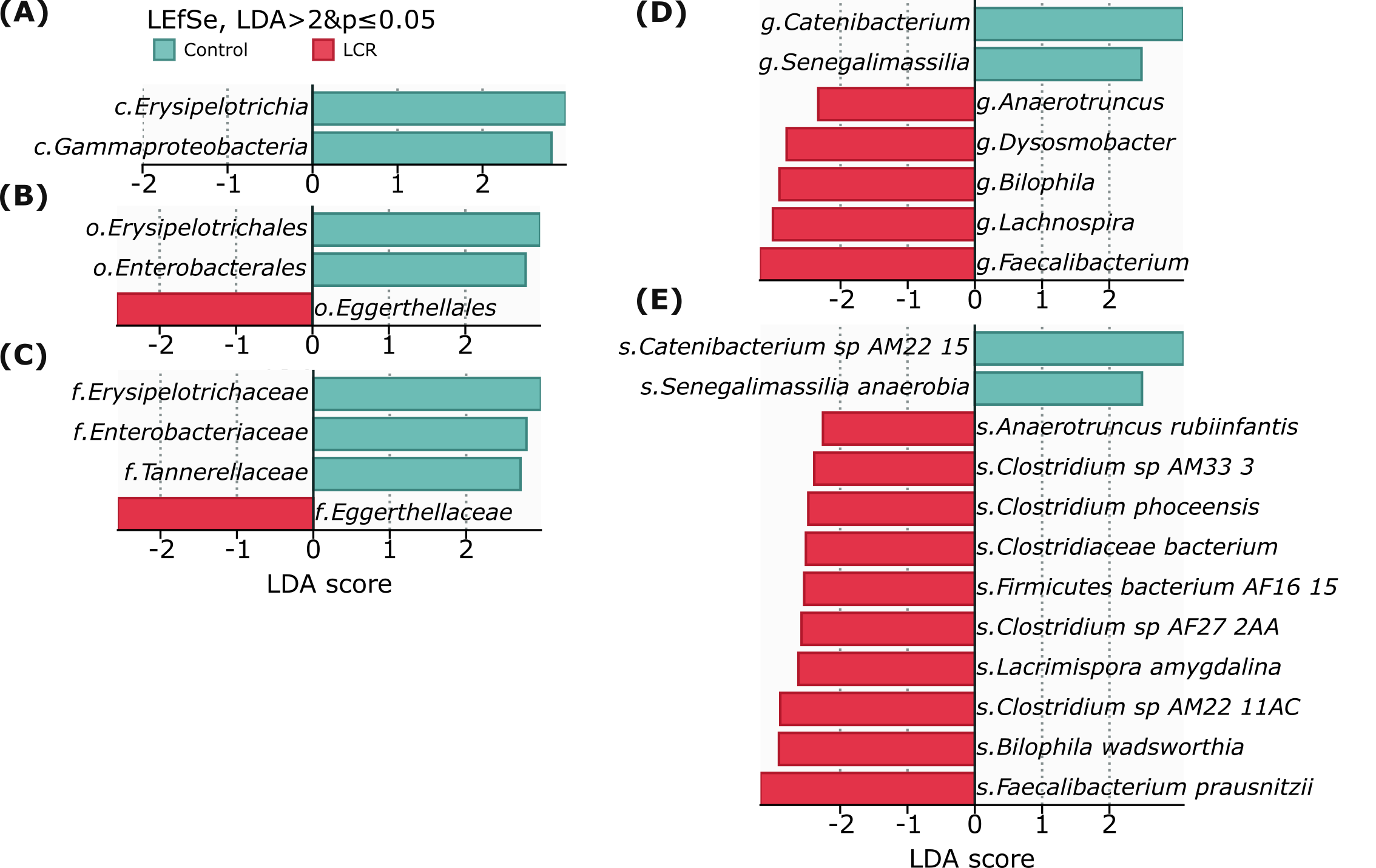

Supplement: Supplementary file 1 [file jcm-14-05097-s001.zip › Suppl figures/Figure S2.png]

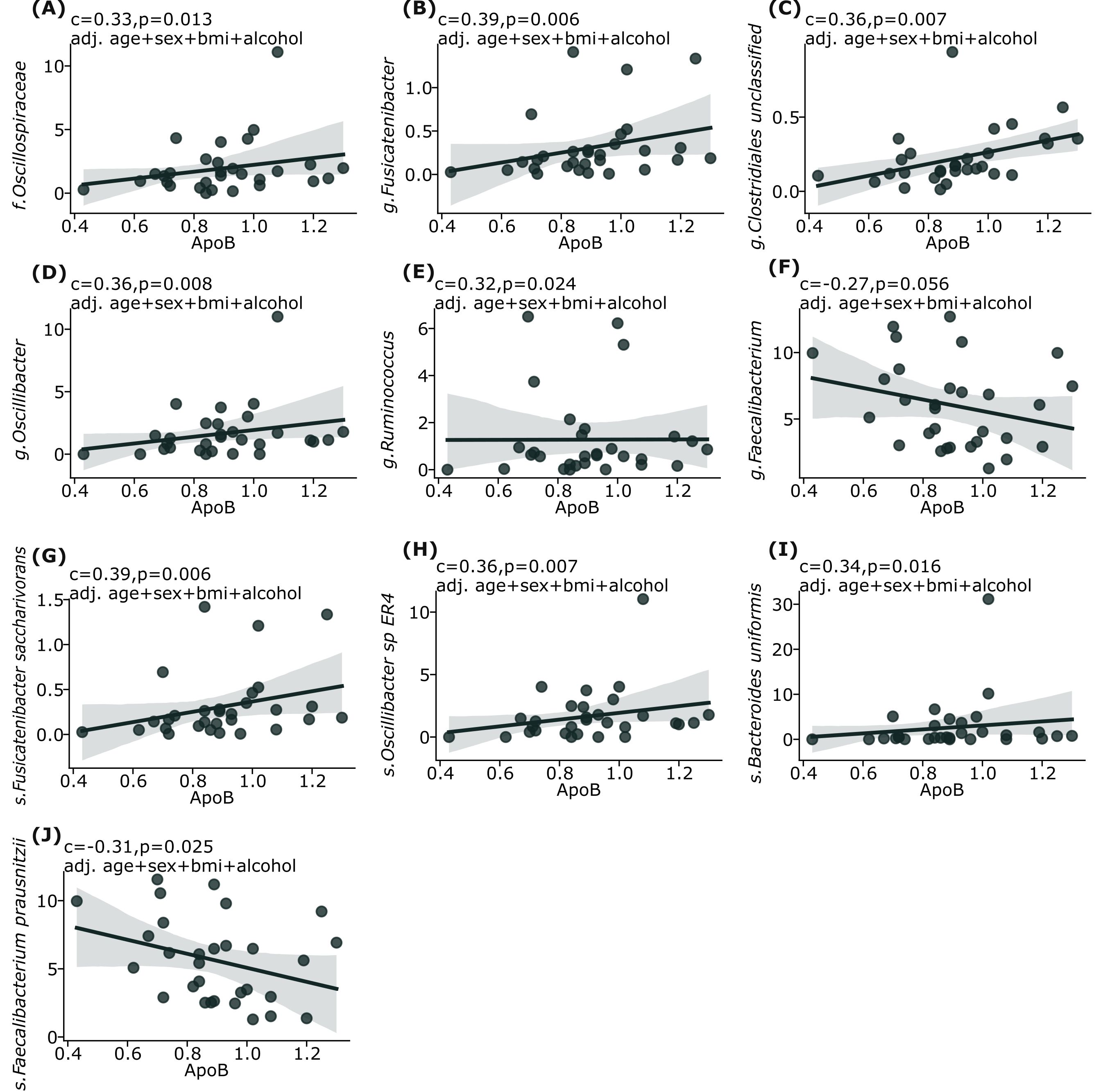

Supplement: Supplementary file 1 [file jcm-14-05097-s001.zip › Suppl figures/Figure S3.png]
